# Cross-reactive antibody against human coronavirus OC43 spike protein correlates with disease severity in COVID-19 patients: a retrospective study

**DOI:** 10.1080/22221751.2021.1905488

**Published:** 2021-04-02

**Authors:** Li Guo, Yeming Wang, Liang Kang, Yongfeng Hu, Linghang Wang, Jingchuan Zhong, Hong Chen, Lili Ren, Xiaoying Gu, Geng Wang, Conghui Wang, Xiaojing Dong, Chao Wu, Lianlian Han, Ying Wang, Guohui Fan, Xiaohui Zou, Haibo Li, Jiuyang Xu, Qi Jin, Bin Cao, Jianwei Wang

**Affiliations:** a NHC Key Laboratory of Systems Biology of Pathogens, Institute of Pathogen Biology, Chinese Academy of Medical Sciences & Peking Union Medical College, Beijing, People’s Republic of China; b Key Laboratory of Respiratory Disease Pathogenomics and Christophe Mérieux Laboratory, Chinese Academy of Medical Sciences & Peking Union Medical College, Beijing, People’s Republic of China; c Department of Pulmonary and Critical Care Medicine, Center of Respiratory Medicine, National Clinical Research Center for Respiratory Diseases, China–Japan Friendship Hospital, Beijing, People’s Republic of China; d Jin Yin-tan Hospital, Wuhan, People’s Republic of China; e Emergency Department of Infectious Diseases of Beijing Ditan Hospital, Capital Medical University, Beijing, People’s Republic of China; f The Second Affiliated Hospital of Harbin Medical University, Harbin, People’s Republic of China; g Institute of Clinical Medical Sciences, China–Japan Friendship Hospital, Beijing, People’s Republic of China; h Institute of Respiratory Medicine, Chinese Academy of Medical Science, Beijing, People’s Republic of China; i Tsinghua University School of Medicine, Beijing, People’s Republic of China; j Department of Respiratory Medicine, Capital Medical University, Beijing, People’s Republic of China

**Keywords:** Cross-reactivity, antibody, HCoV-OC43, SARS-CoV-2, disease severity

## Abstract

Seasonal human coronaviruses (HCoVs) including HCoV-229E, -OC43, -NL63, and -HKU1 widely spread in global human populations. However, the relevance of humoral response against seasonal HCoVs to COVID-19 pathogenesis is elusive. In this study, we profiled the temporal changes of IgG antibody against spike proteins (S-IgG) of SARS-CoV-2 and seasonal HCoVs in 838 plasma samples collected from 344 COVID-19 patients. We tested the antigenic cross-reactivities of S protein between SARS-CoV-2 and seasonal HCoVs and evaluated the correlations between the levels of HCoV-OC43 S-IgG and the disease severity in COVID-19 patients. We found that SARS-CoV-2 S-IgG titres mounted until days 22–28, whereas HCoV-OC43 antibody titres increased until days 15–21 and then plateaued until day 46. However, IgG titres against HCoV-NL63, −229E, and -HKU1 showed no significant increase. A two-way cross-reactivity was identified between SARS-CoV-2 and HCoV-OC43. Neutralizing antibodies against SARS-CoV-2 were not detectable in healthy controls who were positive for HCoV-OC43 S-IgG. HCoV-OC43 S-IgG titres were significantly higher in patients with severe disease than those in mild patients at days 1–21 post symptom onset (PSO). Higher levels of HCoV-OC43 S-IgG were also observed in patients requiring mechanical ventilation. At days 1–10 PSO, HCoV-OC43 S-IgG titres correlated to disease severity in the age group over 60. Our data indicate that there is a correlation between cross-reactive antibody against HCoV-OC43 spike protein and disease severity in COVID-19 patients.

## Introduction

The emergence of coronavirus disease 2019 (COVID1-9), which is caused by severe acute respiratory syndrome coronavirus 2 (SARS-CoV-2), is rapidly spreading around the world and poses an unprecedented public health crisis [1]. As of 10 March 2021, at least 117.3 million infections and 2.6 million deaths have been reported. The manifestation of SARS-CoV-2 infection ranges from asymptomatic to fatal due to substantial heterogeneity in the host responses to the viral infection [2]. Full understanding on the viral pathogenesis is urgently deserved to develop effective intervention to decrease mortality. However, the pathogenesis of SARS-CoV-2 has not been well understood.

The host develops innate, cellular, and humoral immune responses to fight against SARS-CoV-2 infection. Previous studies have shown that dysregulated immune responses play pivotal role in pathogenesis of COVID-19 [3]. Humoral immune response plays a central role in the clearance of virus infection and forms the memory response which helps to prevent virus reinfection. Most infected individuals elicit humoral immune response against SARS-CoV-2, and the virus-specific IgM, IgG, IgA, and neutralizing antibodies (NAbs) can be detected following viral infection [4–6].

Four seasonal human coronaviruses (HCoVs), -NL63, 229E, -OC43, and -HKU-1 have been previously identified prior to the emergence of SARS-COV-2. These viruses typically cause self-limited common colds with mild symptoms in majority. The antibodies against seasonal HCoVs are common in humans due to repeated infections with age [7]. The seroprevalence can reach more than 90% for the four seasonal HCoVs in adults, which is much higher than that in children [7,8]. Interaction between emerging infection and underlying host immunity is very complicated and has drawn attentions on the roles of host immune responses in viral disease. Studies showed that pre-existing antibodies against seasonal influenza is a risk factor for severe diseases and poor outcomes in 2009 pandemic influenza H1N1 patients [9]. Recently, T cell reactivities against SARS-CoV-2 were observed in individuals without exposure history to the virus, indicating possible cross-reactive cellular immunity between seasonal HCoVs and SARS-CoV-2 in human [10]. Sagar’s study showed that recent seasonal coronavirus infection was associated with less severe COVID-19 [11]. Whether there is cross-reactive humoral immunity between seasonal HCoVs and SARS-CoV-2 and its relevance to viral pathogenesis remains elusive.

Antibodies against the nucleocapsid (N) protein and the spike (S) glycoprotein are detected in COVID-19 patients [4,12]. N protein is expressed abundantly during infection which also acts as a strong antigen to elicit immune response during HCoV infection [13]. The S protein, a glycosylated protein which forms a homotrimer to bind the host cell angiotensin-converting enzyme 2 (ACE2) receptor through the receptor binding domain (RBD), is the major target of NAb responses. SARS-CoV-2 and SARS-CoV appear to have antibody cross-reactivities for the N and S proteins [4,14]. Cross-neutralization between the two viruses is a rare event, which suggests that the cross-reactive antibodies may not be protective [14].

To clarify the cross-reactivity between antibodies against SARS-CoV-2 and seasonal HCoVs, we here examined the antigenic cross-reactivity between SARS-CoV-2 and HCoV-OC43. We identified concordant increase of HCoV-OC43 S-IgG with SARS-CoV-2 S-IgG in COVID-19 patients. We found a correlation between cross-reactive HCoV-OC43 S-IgG titre and disease severity in COVID-19 patients. Our findings provide new insights into the pathogenesis of COVID-19.

## Materials and methods

### Patients and plasma samples

In this study, a total of 838 plasma samples from 344 COVID-19 patients were collected from four cohorts in China, including 194 samples from 120 mild/moderate (hereafter “mild”) patients and 644 samples from 224 severe/critical patients including 49 deaths (hereafter “severe”). The definition of mild, moderate, severe, and critical patients followed the Chinese management guideline for COVID-19 (version 6.0) [15].

1. Mild patients: patients with COVID-19 may have non-specific symptoms such as fever, fatigue, cough, anorexia, malaise, muscle pain, sore throat, dyspnoea, nasal congestion, or headache. (2) Moderate patients: adult with pneumonia but no signs of severe pneumonia and SpO2 > 93% on room air. (3) Severe patients: adult with pneumonia but no signs of severe pneumonia and SpO2 ≤ 93% on room air. (4) Critical patients: adult with ARDS or sepsis/septic shock. In this study, mild, and moderate patients are collectively referred to as “mild patients” hereafter, while severe and critical patients are collectively referred to as “severe patients” hereafter.

The first cohort came from Wuhan city during the early phase of the pandemic in January 2020 [4]. The second cohort was recruited from Beijing hospitals in January 2020 [4]. The third cohort was recruited from an outbreak in Harbin, Heilongjiang Province in April 2020 (unpublished data). The plasma samples of the fourth cohort came from a clinical trial on the efficacy of lopinavir–ritonavir in adults hospitalized with severe COVID-19 (LOTUS China) at Wuhan Jinyintan Hospital [16]. Most of the plasma samples from cohort 1, 2, and 4 have been used in previous studies [4,17] The plasma samples were obtained 1–46 days post symptom onset (PSO), with 65 samples at days 1–7 PSO, 258 samples at days 8–14 PSO, 289 samples at days 15–21 PSO, 169 samples at days 22–28 PSO, and 57 samples at days 29–46 PSO. Participant demographics and the number of plasma samples in each cohort are shown in Table S1 and S2. The diagnosis of COVID-19 was confirmed by qPCR assay and lung computed tomography (CT) according to diagnostic guidelines for COVID-19 [18].

A total of 672 throat swab samples were obtained from 257 COVID-19 patients. The throat swab samples were collected simultaneously with the plasma, with 12 throat swab samples (12 patients) from cohort 1, 39 (28 patients) from cohort 2, 25 (25 patients) from cohort 3, and 596 (192 patients) from cohort 4 (Table S3).

Healthy volunteer plasma samples were collected from 278 unexposed healthy individuals before 2019 from 1 to 70 years old in Beijing and Wuhan for regular health check-ups.

All the plasma samples taken from COVID-19 patients and healthy individuals were inactivated at 56°C for 30 min before performing ELISA, indirect immunofluorescence assay (IFA), and microneutralization (MN). ELISA was performed in Biological Safety Level 2 facilities. IFA and MN were performed in Biological Safety Level 3 facilities.

### Plain enzyme-linked immunosorbent assay (ELISA)

We developed plain ELISA protocols for detecting IgG against S proteins of seasonal HCoVs and SARS-CoV-2 as reported elsewhere, which was used to detect IgG against S protein of SARS-CoV-2 [17]. The purified ectodomain of S proteins of HCoV-NL63 (Met1–Pro1296), -229E (Cys16–Trp1115), -OC43 (Met1–Pro1304), -HKU1 (Met1–Pro1295), and SARS-CoV-2 (Val 16–Pro1213), which were expressed in insect cells (Sino Biological, Beijing, China), were used as coating antigens (20 ng/well), respectively. Horseradish peroxidase (HRP)-conjugated goat anti-human IgG (Sigma-Aldrich, St. Louis, MO, USA) diluted as 1:60,000 was used as the secondary antibody. The optimal coating concentration of antigen and optimal plasma dilutions were 20 ng/well and 1:400, respectively, using a checkerboard titration method. To determine the cut-off values for the ELISAs, we determined the mean values and standard deviations (SDs) of negative plasma against SARS-CoV-2, HCoV-NL63, -229E, OC43, and HKU1, respectively. The cut-off values were determined by calculating the mean absorbance at 450 nm of the negative plasma plus 3-fold the SD values [4], which were 0.21, 0.20, 0.20, 0.20, and 0.20 for S-IgG against SARS-CoV-2, HCoV-NL63, -229E, OC43, and HKU1, respectively.

### Competitive ELISA

A competitive ELISA was performed as previously described to evaluate the cross-reactivity between S-IgG against SARS-CoV-2 and seasonal HCoVs [19]. Prior to the SARS-CoV-2 S-IgG ELISA assay, COVID-19 patient plasma samples were absorbed with S proteins of seasonal HCoVs, respectively. In turn, prior to the seasonal HCoVs S-IgG ELISA assay, COVID-19 patients plasma samples were absorbed with SARS-CoV-2 S proteins. For this purpose, an optimal dilution of heterologous S protein (2 μg/mL) was added to a 1:400 dilution of human plasma and incubated for 1 h at 4°C prior to performing the SARS-CoV-2 and seasonal HCoVs S-IgG ELISA assays. The cut-off values of competitive ELISA were the same as those of plain ELISA.

### Detection of HCoV-OC43 RNA in respiratory samples

All the throat swabs were tested by Real-Time Quantitative Reverse Transcription PCR (qRT-PCR) against the membrane protein gene to screen HCoV-OC43 as described previously [20].

### Indirect immunofluorescence assay (IFA)

Vero cells were infected with SARS-CoV-2 at a MOI of 0.1 in 96-well plates for 24 h. The cells were fixed with 4% formaldehyde, permeabilized with 0.5% Triton X-100, and blocked with 5% BSA. The 1:100 diluted human plasma samples were used as the primary antibody. Alexa Fluor 488-labeled anti-human IgG (Life Technologies, Eugene, OR, USA) was used as the secondary antibody. The nuclei were stained with DAPI (Sigma-Aldrich). The images were obtained by scanning with an Operetta High Content Screening system (PerkinElmer, Waltham, MA, USA) and the number of SARS-CoV-2 infected Vero cells and fluorescent intensity were quantified by using the same software.

### Western blot analysis

Purified S protein of HCoV-OC43 and SARS-CoV-2, or SARS-CoV-2 infected Vero cell lysates were separated by 12% sodium dodecyl sulphate polyacrylamide gel electrophoresis (SDS-PAGE) gels and transferred to a nitrocellulose membrane (Pall, Port Washington, NY, USA). Human plasma samples positive for SARS-CoV-2 or HCoV-OC43 were applied to probe S proteins of SARS-CoV-2 or HCoV-OC43. An in-house monoclonal antibody against SARS-CoV-2 S2 subunit were used as positive control. Goat antihuman IRDye Fluor 800-labeled IgG secondary antibody was used at a dilution of 10,000 (Li-Cor, Lincoln, NE, USA). The membranes were scanned by using the Odyssey Infrared Imaging System (Li-Cor).

### Microneutralization assay

A serial two-fold dilution of plasma samples (starting at 1:10) was pre-incubated with SARS-CoV-2 (IPBCAMS-WH-01/2019, no. EPI_ISL_402123) at 100 TCID50 (50% tissue culture infective doses) determined by Vero cells (ATCC, CCL-81). After 2 h of incubation, the virus/plasma mixture was incubated with Vero cells in 96-well plates (Costar). The virus/plasma mixtures were removed after 1 h and fresh growth medium was added to each well. The cytopathic effects were evaluated 5 days after incubation at 37°C in 5% CO_2_. For each plasma dilution, 4 duplicate wells were used. The NAb titres were calculated by using Reed and Muench method [21].

### Cytokine and chemokine measurement

Plasma cytokines and chemokines were measured using Human Cytokine Standard 27-Plex Assays panel and the Bio-Plex 200 system (Bio-Rad, Hercules, CA, USA) for 707 samples according to the manufacturer’s instructions. The plasma samples from four healthy adults were used as controls.

### Ethics approval

The study was approved by the Institutional Review Boards of Wuhan Jinyintan Hospital, Infectious Disease Hospital of Heilongjiang Province and Institute of Pathogen Biology, Chinese Academy of Medical Sciences (KY-2020-02.02, 20200401, IPB-2020-22). Written informed consent was obtained from each healthy volunteer and COVID-19 patients in cohort 4. Written informed consents from the remaining patients were waived in light of the emerging infectious disease of high public health relevance.

### Statistical analysis

Continuous variables were compared with non-parametric test. Paired plasma antibody titres were compared with two-tailed Wilcoxon matched-pairs signed-rank test. Categorical variables were compared with the *χ*^2^ test. Associations between HCoV-OC43 S-IgG titres and disease severity of COVID-19 patients were identified using a Spearman’s rank correlation test. Two-sided *P* < 0.05 was considered to be statistically significant. All statistical analysis was conducted using GraphPad Prism 9.0.0.

## Results

### Clinical characteristics of the patients

The 344 recruited patients were aged from 15–85 (median of 53) years old, with 209 males (Table 1). Major clinical manifestations included fever, cough, dyspnoea, fatigue, sore throat, and muscle pain. Comorbidities were recorded in 191 (55.5%) patients based on the patients’ self-report on admission including hypertension, diabetes, heart disease, chronic respiratory diseases, and chronic kidney disease, etc. (Table 1).

**Table 1.** Demographic and clinical features of COVID-19 patients recruited in this study.

	Total	Mild	Severe (survivor)	Severe (non-survivor)	*P* value
Cases	344	120	175	49	/
					
Age (Years)					
Median (IQR)*	53 (44–64)	47 (38–55)	56 (46–65)	65 (55.5–73)	<0.0001
					
Gender, n (%)					
Male	209 (608)	70 (58.3)	107 (61.1)	33 (67.3)	0.5515
Female	135 (39.2)	50 (41.7)	68 (38.9)	16 (32.7)	0.5515
					
Underlying diseases, n (%)					
Yes	191 (55.5)	40 (33.3)	111 (63.4)	40 (81.6)	<0.0001
Hypertension	101 (29.4)	17 (14.2)	64 (36.6)	20 (40.8)	<0.0001
Diabetes	34 (9.9)	3 (2.5)	23 (13.1)	8 (16.3)	0.00286
Heart disease	33 (9.6)	11 (9.2)	16 (9.1)	6 (12.2)	0.7932
Cerebrovascular disease	18 (5.2)	5 (4.2)	9 (5.1)	4 (8.2)	0.5694
Chronic kidney disease	11 (3.2)	2 (1.7)	6 (3.4)	3 (6.1)	0.3178
Malignancies	8(2.3)	1 (0.83)	5 (2.9)	0 (0.0)	0.2572
Chronic respiratory diseases	6 (1.7)	2 (1.7)	3 (1.7)	1 (2.0)	0.985
					
Symptoms, n (%)					
Fever	305 (88.7)	98 (81.7)	160 (91.4)	47 (95.9)	<0.0001
Dyspnoea	68 (19.8)	22 (18.3)	25 (14.3)	21 (42.9)	<0.0001
Fatigue	82 (23.8)	19 (15.8)	44 (25.1)	19 (38.8)	0.0054
Cough	274 (79.7)	85 (70.8)	148 (84.6)	41 (83.7)	0.0119
Pharyngalgia	28 (8.1)	8 (6.7)	11 (6.3)	9 (18.4)	0.0231
Muscle pain	57 (16.6)	19 (15.8)	26 (14.9)	12 (24.5)	0.2669
Headache	34 (9.9)	14 (11.7)	14 (8.0)	6 (12.2)	0.4887
Diarrhea	10 (2.9)	3 (2.5)	6 (3.4)	1 (2.0)	0.8314

Note: *P* values were calculated by Kruskal–Wallis test or *χ*^2^ test as appropriate. *IQR, interquartile range.

### Kinetic patterns of IgG against S protein of SARS-CoV-2 and seasonal HCoVs in COVID-19 patients

**Figure 1.**
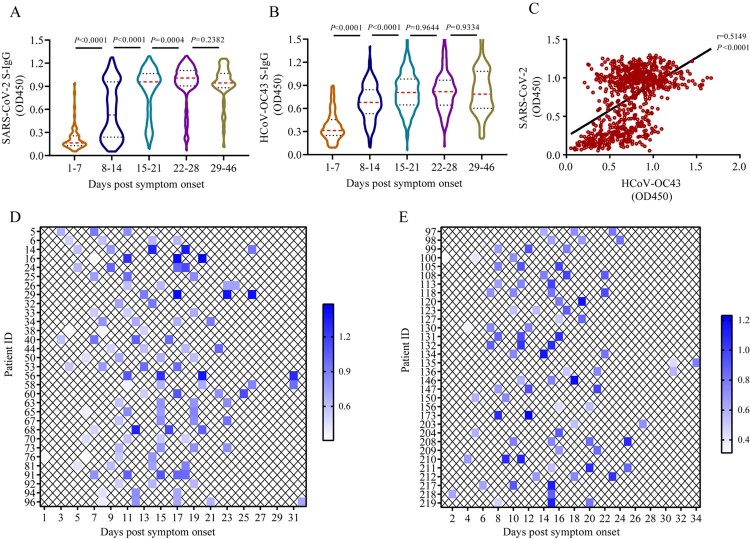
Temporal profiles of IgG antibodies against S proteins of SARS-CoV-2 and HCoV-OC43.(A–B) Dynamic changes of SARS-CoV-2 S-IgG (A) and HCoV-OC43 S-IgG (B) levels in COVID-19 patient’ plasma samples over time post symptom onset measured by the plain ELISA assays (for details, see Materialans and Methods). Red and black dotted lines in violin denote the median and interquartile range of antibody titres, respectively. Non-parametric Mann–Whitney test was used for comparison of antibody titres. (C) Associations between the levels of HCoV-OC43 S-IgG and SARS-CoV-2 S-IgG in COVID-19 patient’s plasma samples. The correlation were assessed by Spearman’s rank correlation test. (D–E) Kinetics of HCoV-OC43 S-IgG levels in COVID-19 patients who had 2–5 consecutive plasma samples measured by plain ELISA assay. Each key presents the OD450 of each plasma at indicated time point.

We found that IgG against S protein (S-IgG) of SARS-CoV-2 developed as early as day 2 PSO and mounted over time until days 22–28 PSO. Similar trends were also observed in HCoV-OC43 S-IgG, but not in HCoV-NL63, −229E, and -HKU1 in the plain ELISA assays (Figure 1(A–B), Figures S1A–S1C). The HCoV-OC43 S-IgG levels increased from days 1–7 (median, 0.31) to days 8–14 (median, 0.68; *P* < 0.0001), continued to increase until days 15–21 (median, 0.81; *P* < 0.0001), and finally plateaued until days 46 PSO (Figure 1(B)). To confirm this observation, we further analysed 17 patients who had the consecutive samples collected at days 1–7 and days 8 –14 and 59 patients who had consecutive samples collected at days 8–14, 15–21, and 22–28. The data also showed that HCoV-OC43 S-IgG titres increased in these consecutive samples at days 1–7, 8–14, and 15–21 (Wilcoxon matched-pairs signed-rank test, *P* = 0.002, and <0.0001, respectively) (Figures S2A-S2B). The levels of HCoV-OC43 S-IgG showed a positive correlation with those of SARS-CoV-2 S-IgG (Spearman *r* = 0.5149, *P* < 0.0001) (Figure 1(C)). However, the SARS-CoV-2 S-IgG level was not found to be significantly associated with those of HCoV-NL63 S-IgG (Spearman *r* = 0.04109, *P* = 0.3602) and HCoV-229E S-IgG (Spearman *r* = 0.03480, *P* = 0.4384), but a slight correlation with those of HCoV-HKU1 S-IgG (*r* = 0.3449, *P* < 0.0001) (**Figures S1D–S1F**). Among 178 cases who had 2–5 consecutive plasma samples from cohort 4, HCoV-OC43 S-IgG OD value increased by >20% in 63 cases (35.4%) in the plain ELISA assay (Figure 1(D,E)), while SARS-CoV-2 S-IgG levels increased >20% in 78 cases (43.8%). These results indicate concomitantly temporal patterns of HCoV-OC43 S-IgG and SARS-CoV-2 S-IgG in COVID-19 patients. A total of 68 COVID-19 patients (84 plasma samples) were negative for SARS-CoV-2 S-IgG. The reason may be due to the SARS-CoV-2 S-IgG had not developed when the samples were collected. And a total of 10 COVID-19 patients (13 plasma samples) were negative for HCoV-OC43 S-IgG.

To exclude the possibility that the increase of the HCoV-OC43 S-IgG titres was resulted from the co-infection of HCoV-OC43 with SARS-CoV-2, we screened the HCoV-OC43 infection using all obtained 672 throat swabs from 257 COVID-19 patients by qRT-PCR. As expected, HCoV-OC43 was not detected in any of the samples (Figure S3).

### Two-way cross-reactivities between SARS-CoV-2 and HCoV-OC43

**Figure 2.**
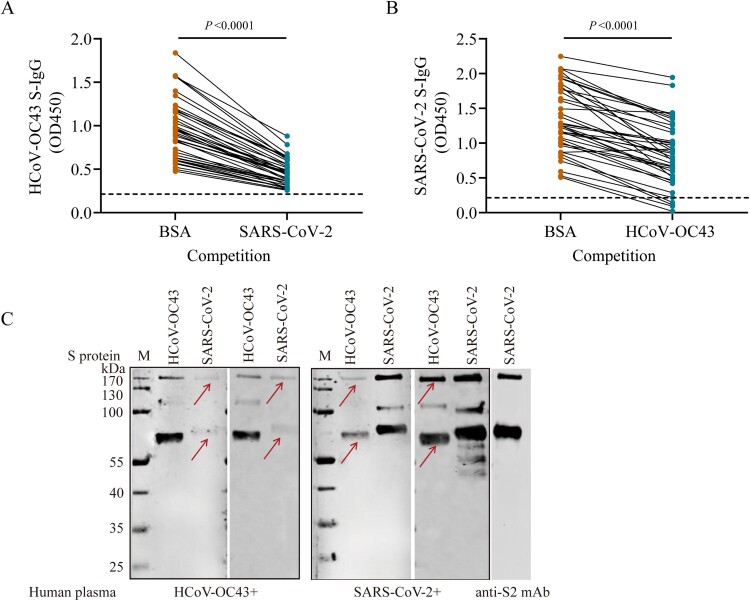
Two-way antigenic cross-reactivities between SARS-CoV-2 and HCoV-OC43. (A) HCoV-OC43 S-IgG levels in plasma samples taken from COVID-19 patients (*n* = 50) after competition by 0.5% bovine serum albumin (BSA) or SARS-CoV-2 S protein, respectively. (B) SARS-CoV-2 S-IgG levels in plasma samples taken from COVID-19 patients (*n* = 50) after competition using 0.5% BSA or HCoV-OC43 S protein, respectively. (C) Western blot analysis to determine the cross-reactivity between S proteins of SARS-CoV-2 and HCoV-OC43. Two human plasma samples positive for HCoV-OC43 S-IgG and two human plasma samples positive for SARS-CoV-2 S-IgG were used to probe the S proteins of SARS-CoV-2 and HCoV-OC43, respectively. Plasma samples were diluted at 1:400 and S protein of HCoV-OC43 and SARS-CoV-2 were loaded at 250 ng/well, respectively. A monoclonal antibody against SARS-CoV-2 S2 subunit (Anti-S2 mAb) was used as positive control. The red arrows indicate ectodomain and S2 subunit of S protein, respectively. Two-tailed Wilcoxon matched-pairs signed-rank test was used for antibody level comparison.

To explore whether the increasing antibody titres against HCoV-OC43-S protein came from the cross-reactivities between HCoV-OC43 and SARS-CoV-2, we performed a competitive ELISA assay using 50 plasma samples taken from COVID-19 patients who had higher absorbance values for HCoVs from cohorts 1 and 4. We first assessed the cross-reactivity between HCoV-OC43 S-IgG and SARS-CoV-2 S protein. We found that HCoV-OC43 S-IgG titres were significantly decreased (Wilcoxon matched-pairs signed-rank test, *P* < 0.0001) when the COVID-19 patients’ plasma were pre-incubated with SARS-CoV-2 S proteins (Figure 2(A)). In turn, we then assessed the cross-reactivity between SARS-CoV-2 S-IgG and HCoV-OC43 S protein. We found that SARS-CoV-2 S-IgG absorbance values decreased (Wilcoxon matched-pairs signed-rank test, *P*<0.0001) when the COVID-19 patients’ plasma were pre-treated with the HCoV-OC43 S protein (Figure 2(B)). These data suggest that some individuals infected with SARS-CoV-2 could produce cross-reactive antibody responses to HCoV-OC43. The cross-reactivities were further verified by Western blot with HCoV-OC43-positive plasma from unexposed healthy individuals which were collected before 2019 and SARS-CoV-2 S protein, as well as SARS-CoV-2 positive plasma from confirmed COVID-19 patients and HCoV-OC43 S protein. The specific bands of both S protein and S2 subunit were detected (Figure 2(C)). The low level of SARS-CoV-2 S-IgG was detected in 48% (48/100) unexposed adult plasma and in 9.9% (19/192) unexposed child plasma which were collected before 2019 (Figure S4). Collectively, these data suggest a two-way cross-reactivity between the S proteins of SARS-CoV-2 and HCoV-OC43, indicating the presence of cross-reactive epitope(s) in the S proteins between SARS-CoV-2 and HCoV-OC43.

**Figure 3.**
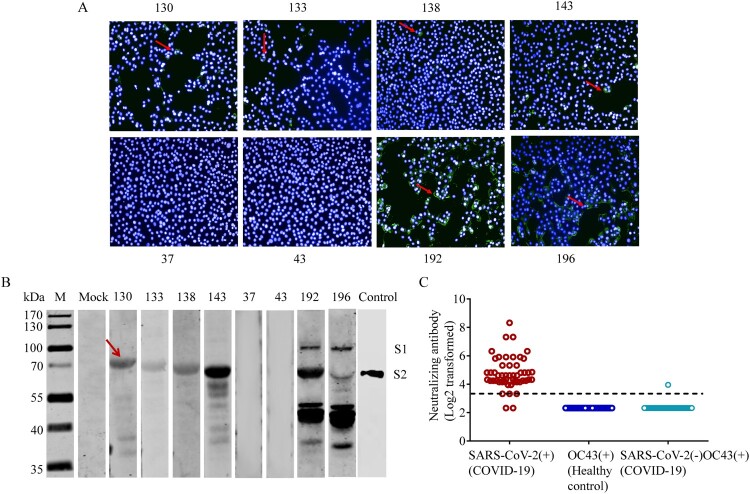
Cross-reactivities between SARS-CoV-2 and HCoV-OC43 S-IgG. (A–B) Cross-reactivities between HCoV-OC43 S-IgG and SARS-CoV-2 in indirect immunofluorescence assays (IFAs) (A) and Western blot assays (B). Vero cells infected with SARS-CoV-2 at a MOI of 0.1 were probed with plasma positive for HCoV-OC43 S-IgG from healthy donors (sample ID: 130, 133, 138, and 143). Plasma samples negative for HCoV-OC43 antibodies were used as negative controls (sample ID: 37 and 43). Plasma from COVID-19 patients were used as positive controls (sample ID: 192, 196). Control (in panel B): The S2 subunit were verified using a monoclonal antibody against SARS-CoV-2 S2 subunit (Control). The red arrows in panel (A) indicate Vero cells that can reacted with plasma samples positive for HCoV-OC43 or SARS-CoV-2. The red arrows in panel (B) indicate the S2 subunit of S protein. (C) Cross-neutralization between SARS-CoV-2 and HCoV-OC43 S-IgG. Neutralizing antibodies (NAbs) were measured using HCoV-OC43 S-IgG positive plasma from 50 unexposed healthy controls, and HCoV-OC43 S-IgG positive but SARS-CoV-2 S-IgG negative plasma from 50 COVID-19 patients by microneutralization assay with a SARS-CoV-2 isolate (IPBCAMS-WH-01/2019 strain). SARS-CoV-2 S-IgG positive plasma samples from 50 COVID-19 patients were used as positive control. Dashed line represents the cut-off value of NAb titres.

Cross-reactivities were further verified by IFA using SARS-CoV-2-infected cells with plasma samples positive for HCoV-OC43 collected from unexposed healthy volunteers before 2019 (sample ID: 130, 133, 138, and 143), plasma samples negative for HCoV-OC43 antibodies as negative control (sample ID: 37 and 43), and plasma samples from COVID-19 patients as positive control (sample ID: 192, 196) (Figure 3(A)). In general, followed by SARS-CoV-2 infections, the intensities of IFA in Vero cells pretreated with HCoV-OC43-positive plasma were about 10-20-fold lower than those pretreat with SARS-Cov-2 -positive plasma (Figure 3(A)). Further, the SARS-CoV-2 infected cell lysates were subjected to Western blot probed with these plasma samples and showed immunoreactivity with COVID-19 patient plasma at both the S1 and S2 subunits. The SARS-CoV-2-infected cell lysates also reacted with unexposed human plasma positive for HCoV-OC43 at S2 subunit (Figure 3(B)). The S2 subunit was verified using a monoclonal antibody (mAb) against SARS-CoV-2 S2 subunit (Figure 3(B)). These findings suggest that the cross-reactivities between SARS-CoV-2 and HCoV-OC43 are mainly mediated by the S2 subunit.

We also evaluated the cross-reactivities between the S proteins of SARS-CoV-2 and the other three seasonal HCoVs, HCoV-NL63, -229E, and -HKU1. We found that the cross-reactivities of S proteins between HCoV-NL63 and SARS-CoV-2, HCoV-229E and SARS-CoV-2, as well as HCoV-HKU1 and SARS-CoV-2 were present in a few persons, respectively (Figures S5 A-F). The cross-reactivity between SARS-CoV-2 and alpha-coronaviruses deserve further studies.

### No cross-neutralizing antibody responses between plasma containing HCoV-OC43 S-IgG and SARS-CoV-2

As S protein is the major immunogen which can elicit NAbs, we then carried out microneutralization assays to determine whether the HCoV-OC43 S protein could induce antibodies which can functionally neutralize SARS-CoV-2. We first verified the presence of HCoV-OC43 S-IgG using unexposed healthy controls which were collected before 2019, including 192 children and 86 adults. The results showed that the titres of HCoV-OC43 S-IgG in adults are higher than those in children, and there is no difference between the HCoV-OC43 S-IgG level of COVID-19 patients in acute phase (days 1–10 PSO) and that of the unexposed adults (Mann–Whitney test, *P* = 0.2078) (Figure S6). We then tested cross-neutralization using 50 unexposed healthy controls positive for HCoV-OC43 S-IgG. Among these positive controls of 50 COVID-19 patients, nearly all plasma samples could neutralize SARS-CoV-2 with NAb titres ranging from 1:10 to 1:320 except two plasma samples that came from non-survivors (Figure 3(C)). However, despite the existence of cross-reactivities, no cross-neutralization was detected in healthy volunteer plasma samples (Figure 3(C)). Further, we performed microneutralization assay using 50 plasma samples taken from COVID-19 patients that were HCoV-OC43 S-IgG positive but SARS-CoV-2 S-IgG negative. The results showed that only one sample presented neutralizing activity at a very low titre, but no neutralization was detected in other samples **(**Figure 3(C)). This positive plasma sample was collected on day 18 PSO from a female 48-year-old COVID-19 patient with a mild symptom including fever and cough. These results indicate that cross-neutralization between HCoV-OC43 positive plasma and SARS-CoV-2 may be rare.

### Higher HCoV-OC43 antibody titres are correlated with disease severity

**Figure 4.**
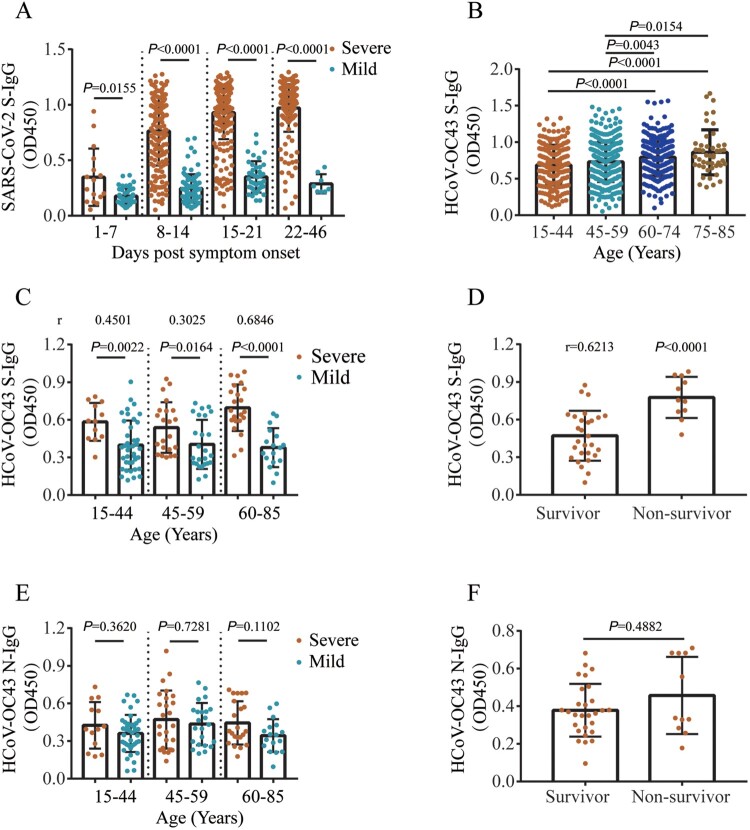
Cross-reactive HCoV-OC43 antibody titres correlate with disease severity in COVID-19 patients. (A) Dynamic changes of SARS-CoV-2 S-IgG levels in mild and severe COVID-19 patients. (B) HCoV-OC43 S-IgG levels in COVID-19 patients in different age groups over time post symptom onset (PSO). (C) The correlation between the HCoV-OC43 S-IgG titres and disease severity (mild and severe) in different age groups. (D) The correlation between HCoV-OC43 S-IgG titres and clinical outcome (survivor and non-survivor) in patients aged 60–85 yearsat days 1–10 PSO. (E) The correlation between HCoV-OC43 N-IgG titres and disease severity (mild and severe) in different age groups. (F) The correlation between HCoV-OC43 N-IgG titres and clinical outcome (survivor and non-survivor) in patients aged 60–85 years at days 1–10 PSO. All the antibody titres were analysed using ELISA assays and shown as mean with SD. Non-parametric Mann–Whitney test was used for comparison of antibody titres. The correlation were assessed by Spearman’s rank correlation test.

We then explore the relationship between the cross-reactive antibodies and disease severity in COVID-19 patients. Higher titres of SARS-CoV-2 S-IgG were found in severe COVID-19 cases at days 1–46 PSO (Mann–Whitney test, *P* < 0.0001) compared with mild cases (Figure 4(A)). ELISA analysis showed that the IgG titres against HCoV-OC43 S protein increased with age in COVID-19 patients (Mann–Whitney test, *P* < 0.01) (Figure 4(B)). Compared with mild cases, higher titres of HCoV-OC43 S-IgG were found in severe cases at days 1–7, 8–14, and 15–21 PSO (Mann–Whitney test, *P* < 0.0001, *P* < 0.0001, *P* = 0.0027, respectively), respectively (Figure S7A). Patients who required mechanical ventilation also had higher HCoV-OC43 S-IgG titres (Mann–Whitney test, *P* < 0.001) than those in patients who did not require (Figure S7B). Moreover, severe patients had a positive correlation with HCoV-OC43 S-IgG titres in the age groups 15–44 (Spearman correlation test, *r* = 0.4501, 95% confidence interval [CI], 0.1968–0.647, *P* = 0.0007), 45–59 (*r* = 0.3025, 95% CI, 0.1115–0.5463, *P* = 0.0366), and 60–85 (*r* = 0.6846, 95% CI, 0.4632–0.8255, *P* < 0.0001) at days 1–10 PSO compared with mild cases (Figure 4(C)). Of note, antibody titres in non-survivor were higher (Mann–Whitney test, *P* < 0.0001) than in those survivors aged 60–85 years old at days 1–10 PSO (Figure 4(D)). However, no significant HCoV-OC43 N-IgG titre difference (Mann–Whitney test, *P* > 0.05) was found between mild and severe patients (Figure 4(E)), and between survivor and non-survivor among patients over 60 years old (Figure 4(F)) at days 1–10 PSO. These results suggest that higher cross-reactive HCoV-OC43 S-IgG antibody titres are associated with disease severity in COVID-19 patients, and that the cross-reactive HCoV-OC43 antibody level can be a risk factor for clinical outcomes of COVID-19 patients.

**Figure 5:**
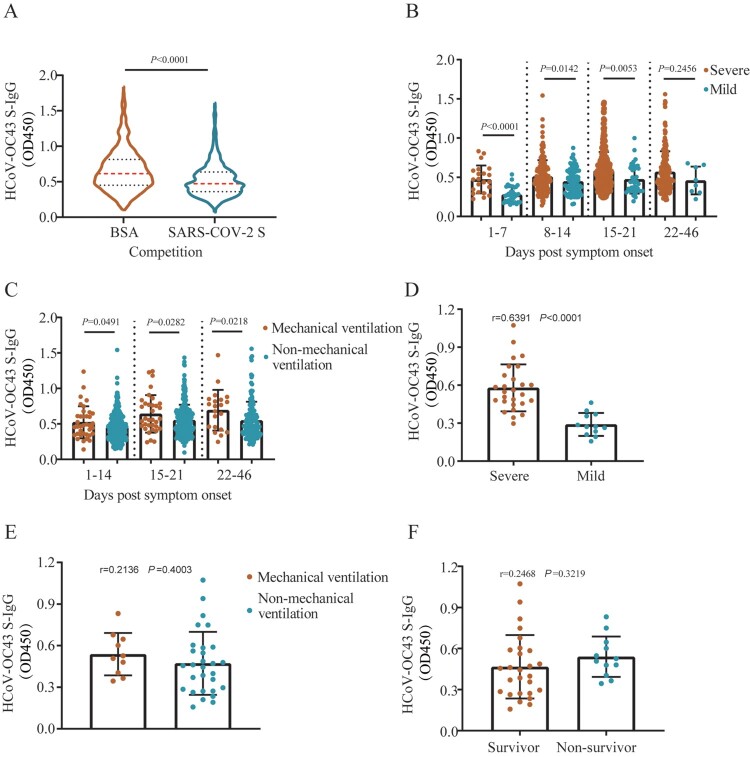
. Correlation between HCoV-OC43 antibody titres and disease severity in COVID-19 patients’ plasma after SARS-CoV-2 S protein competition. (A) HCoV-OC43 S-IgG antibody levels in plasma samples taken from COVID-19 patients pretreated with 0.5% bovine serum albumin (BSA) or SARS-CoV-2 S protein, respectively. Red and black dotted lines in violin denote the median and interquartile range of antibody titres, respectively. Two-tailed Wilcoxon matched-pairs signed-rank test was used for comparison of antibody titres. (B–C) HCoV-OC43-S-IgG levels in patients with mild and severe symptom (B), in patients with mechanical ventilation and non-mechanical ventilation (C) over time post symptom onset after competition using SARS-CoV-2 S protein. Non-parametric Mann-Whitney test was used for comparison of antibody titres. (D–F) The correlation between HCoV-OC43 S-IgG titres and disease severity (mild and severe) (D), mechanical ventilation and non-mechanical ventilation (E), survivor and non-survivor (F) in patients aged 60–85 years at days 1–10 post symptom onset (PSO) after competition using SARS-CoV-2 S protein. The antibody titres in (B–F) were shown as mean with SD. The correlation was assessed by Spearman’s rank correlation test.

To further clarify the role cross-reactive S-IgG in pathogenesis, HCoV-OC43 S-IgG competitive ELISA analysis was performed using plasma collected from 790 COVID-19 patients. The results showed that HCoV-OC43 S-IgG titres in patients’ plasma pre-incubated with SARS-CoV-2 S protein were significantly decreased (Wilcoxon matched-pairs signed-rank test, *P* < 0.0001) compared with those pre-incubated with 0.5% bovine serum albumin (BSA) (Figure 5(A)). HCoV-OC43 S-IgG titres were still higher in severe cases at days 1–7, 8–14, and 15–21 PSO (Mann–Whitney test, *P* < 0.0001, *P* = 0.0142, *P* = 0.0053, respectively) compared with those in mild cases, respectively (Figure 5(B)). Patients who required mechanical ventilation had higher HCoV-OC43 S-IgG titres (Mann–Whitney test, *P* < 0.05) than in those who did not require (Figure 5(C)). HCoV-OC43 S-IgG antibody titres had a positive correlation with severe patients in the age group 60–85 (Spearman *r *= 0.6391, 95% CI, 0.4099–0.7965, *P* < 0.0001) at days 1–10 PSO compared with that of mild cases (Figure 5(D)). However, we did not find the correlations between ventilation requirement and HCoV-OC43 S-IgG titres (Spearman *r* = 0.2136, 95% CI, −0.1185–0.5032, *P* = 0.4003), as well as mortality and HCoV-OC43 S-IgG titres (Spearman r=0.2468, 95% CI, −0.0841–0.5287, *P* = 0.3219) in age group 60–85 at days 1–10 PSO (Figure 5(E,F)).

In addition, the plasma concentrations of IL-1β, IL-5, IL-7, IL-8, MIP-1β, RANTES, and TNFα were higher in severe patients compared with those of in mild patients (data not shown). As shown in Table 2, there are positive correlations between the titres HCoV-OC43 S-IgG and plasma concentration of cytokines and chemokines, including IL-1β, IL-5, IL-7, IL-8, MIP-1β, RANTES and TNFα. These results suggest high HCoV-OC43 S-IgG levels in COVID-19 patients are related with system inflammatory responses.

**Table 2.** Linear correlation between HCoV-OC43 S-IgG antibody levels and plasma concentrations of cytokines and chemokines.

Cytokines/Chemokines	Slope	95% CI	*P* value
IL1β	2.596 ± 1.098	0.4398–4.752	0.0184
IL5	33.78 ± 11.22	11.74–55.81	0.0027
IL7	3.973 ± 1.878	0.2854–7.66	0.0348
IL8	17.09 ± 4.334	8.579–25.6	<0.001
MIP-1β	113 ± 17.5	78.67– 147.4	<0.001
RANTES	1607 ± 524.6	577.1–2637	0.0023
TNFα	19.06 ± 6.43	6.435–31.69	0.0031

## Discussion

In this study, we evaluated the temporal kinetics of S-IgG responses of SARS-CoV-2 and seasonal HCoVs in COVID-19 patients. Although we identified a two-way cross-reactivity between SARS-CoV-2 and HCoV-OC43 S proteins, the cross-reactive S-IgG against HCoV-OC43 did not functionally neutralize SARS-CoV-2. HCoV-OC43 S-IgG titres were significantly higher in patients with severe disease than those in mild patients at days 1–21 PSO. Higher levels of HCoV-OC43 S-IgG were also observed in patients requiring mechanical ventilation. There is a positive correlation between HCoV-OC43 S-IgG titres and disease severity at days 1–10 PSO in elderly COVID-19 patients over 60 years old.

Our data showed that SARS-CoV-2 S-IgG are elicited in the majority of COVID-19 patients, with the antibody levels increasing over time of the infection. The temporal changes of antibody titres and antibody positive rates coincide with those in other studies [4,5,22]. We found a significant boost of HCoV-OC43 S-IgG in COVID-19 patients over the course of disease. Titres of HCoV-OC43 S-IgG were correlated with those against SARS-CoV-2 in COVID-19 patients, which indicates that primary infection of SARS-CoV-2 can lead to a heterosubtypic HCoV-OC43 S antibody response. A similar heterosubtypic antibody boost has been observed between H7N9 and H3N2 influenza viruses [23]. Western blot analysis indicates that the S2 subunit of the S protein mediates the cross-reactivities. The results of IFA and the Western blot analysis suggest that the S protein carries common epitope(s) between SARS-CoV-2 and HCoV-OC43 which causes the cross-reactivity. Recently, Ng KW et.al. detected SARS-CoV-2 S–reactive antibodies in SARS-CoV-2–uninfected donors and the S–reactive antibodies predominantly target the S2 subunit [24]. Further studies are needed to map the epitope(s) precisely and to evaluate the role of such epitope(s) in diagnostic and vaccine development.

To exclude the possibility that the concomitant increase of HCoV-OC43 S-IgG with SARS-CoV-2 S-IgG may be due to the co-infection with HCoV-OC43 in COVID-19 patients, we tested the co-infection of HCoV-OC43 in 257 out of 344 studied patients using the corresponding respiratory samples by qRT-PCR. As expected, HCoV-OC43 was not detected in any of the samples. To date, studies on COVID-19 in detection of co-infections with other respiratory viruses are limited. Kim et al found that of 116 specimens positive for SARS-CoV-2, 5 (4.3%) were positive for non–SARS-CoV-2 coronaviruses [25]. The study of Nowak et al showed that co-infection was found in 36 (3%) among 1204 patients who were positive for SARS-CoV-2. Non-SARS-CoV-2 coronaviruses (7 for HCOV-NL63, 5 for HCoV-HKU1, 4 for HCoV-229E, and 1 for HCoV- OC43) were the most common concurrent respiratory viruses [26]. Studies in China showed that the frequencies of seasonal HCoV detected in patients with respiratory infections were relatively low [27]. Recent reports also showed that the SARS-CoV-2-positive patients co-infected with other respiratory viruses were very low in China and no seasonal HCoVs were co-detected with SARS-CoV-2 [28,29]. Our data verified that the concomitantly increase of HCoV-OC43 S-IgG in COVID-19 patients may not be elicited by co-infection of HCoV-OC43.

It was found that some healthy individuals without SARS-CoV-2 infection possess T cells capable of recognizing SARS-CoV-2, which may be due to cross-reactive responses between SARS-CoV-2 and previous exposures to seasonal HCoVs [10]. However, the effects of the cross-reactivity on COVID-19 patients were not addressed in these studies. It is possible that cross-reactive T cells have a protective role by helping B cells to accelerate the production of SARS-CoV-2 specific antibodies. On the other hand, it is also possible that pre-existing immunity might be detrimental in the disease progression of COVID-19. In our study, COVID-19 patients with severe symptoms and elderly over 60 years old had higher levels of HCoV-OC43 S-IgG but not N-IgG. Relative absence of severe SARS-CoV-2 infection has been found in children [30,31]; while the proportion of COVID-19-associated hospitalization elevates with age [32]. Considering that children on average have been less exposed to HCoVs infections in their lifetime than adults and children carry a much lower level of antibodies against HCoV-OC43 than adults [33], whether the absence or the lower level of underlying HCoV-OC43 S-IgG contributes to the better prognosis in paediatric COVID-19 patients warrants further studies. A study showed that the sera from some SARS-CoV-2–uninfected individuals neutralized SARS-CoV-2 and SARS-CoV-2 S pseudotypes [24]. Thus, it is important that the effect of preexisting seasonal HCoV antibodies on the course of SARS-CoV-2 infection should be fully delineated and studied.

Previous studies have shown that the antibody titre against N or S protein was higher among severe COVID-19 patients than those of mild patients [5,34]. In this study, we found that the levels of SARS-CoV-2 and HCoV-OC43 S-IgG titres were both higher in patients with severe disease than in those with mild disease progression. Many factors, such as innate and adaptive immune responses, aging, sex, and comorbidities, contribute to the outcome of the COVID-19. To evaluate the real role of HCoV-OC43 S-IgG in pathogenesis, we performed HCoV-OC43 S-IgG ELISA pre-absorbed the plasma from COVID-19 patients with SARS-CoV-2 S protein. The data still showed that HCoV-OC43 S-IgG titres were high in severe cases and patients who required mechanical ventilation. Moreover, HCoV-OC43 S-IgG antibody titres presented a positive correlation with severe patients in the elderly over 60 years old at days 1–10 PSO. This observation excludes that the correlation between cross-reactive HCoV-OC43 S-IgG level and disease severity is solely caused by SARS-CoV-2 S-IgG.

The mechanism responsible for the pathogenic role of cross-reactive antibodies between SARS-CoV-2 and HCoV-OC43 is unclear. Studies have shown that cross-reactive antibodies with lower avidity may result in pathogenic but not protective effects through forming antigen–antibody immune complex, which have been exemplified by the studies on influenza virus [9] and dengue virus (DENV) [35]. The mechanism may involve antibody-dependent enhancement (ADE) of infection triggered by non-protective antibody responses as reported in other coronaviruses, such as SARS-CoV and MERS-CoV [36, 37], and also DENV, HIV, and Ebola virus [38]. The pre-existing cross-reactive antibodies at specific concentrations bind virions of the subsequent infecting virus. These immune complexes recognize Fcγ receptors of immune cells, resulting in the local activation of complement, macrophages, and dendritic cells which can produce a variety of cytokines (“cytokine storms”). Whether cross-reactive HCoV-OC43 antibodies form immune complex or contribute to the ADE of SARS-CoV-2 are unclear. Our data further showed the positive correlation between HCoV-OC43 S-IgG titre and the concentration of a variety of plasma cytokines and chemokines. Cytokine storm can trigger a violent inflammatory response, which contributes to acute respiratory distress syndrome (ARDS), multiple organ failure, and finally death of the COVID-19 patients. Further studies are warranted to clarify how the cross-reactive HCoV-OC43 S-IgG affects COVID-19 pathogenesis.

Our observations were are contradict with Sagar’s study [11], where they reported that recent seasonal coronavirus infection is protective against severe COVID-19 outcomes. The discrepancy may be attributed to the different study designs. Sagar’s study retrospectively examined the medical record of COVID-19 patients who had previously been assessed by nucleic acids for four seasonal HCoVs along with 16 other pathogens. Analysis showed that the patients with a recently documented seasonal HCoVs infection had less severe COVID-19 illness. However, the antibody levels against seasonal HCoVs in these patients were not determined in that study. Moreover, the comprehensive effects of accumulated antibodies against seasonal coronaviruses were not assessed. The absence of seasonal HCoVs nucleic acids at some time points does not preclude the HCoVs antibody presence in these individuals. In our study, we evaluated antibody levels against HCoV-OC43 S proteins and the correlation between cross-reactive antibody level against HCoV-OC43 and disease severity in COVID-19 patients at days 1–10 PSO (which stands for the acute phase of infection), which may comprehensively inform the role of previous exposures of seasonal coronaviruses in COVID-19 pathogenesis.

Our study has several limitations. First, the number of subjects was not large. Prospective cohorts with longitudinal data will help to refine the assessment of the relationship between the level of cross-reactive HCoV-OC43 S-IgG and disease severity in COVID-19 patients. Second, the collection of the plasma samples of COVID-19 patients used in this study was limited in China. We hope that plasma samples from other countries would help to confirm our findings. Thirdly, we did not define the cut-off value of HCoV-OC43 S-IgG for predicting the disease severity. Forth, the structure of the expressed peptides has not been comprehensively characterized.

In summary, we confirmed a two-way antigenic cross-reactivity between the SARS-CoV-2 and HCoV-OC43 S proteins. Our data indicate that cross-reactive HCoV-OC43 S-IgG does not neutralize SARS-CoV-2 but associated with disease severity in COVID-19 patients. These findings inform the understanding on the pathological processes during SARS-CoV-2 infections and factors may influence prognosis.

## Supplementary Material

Supplementary_materials.docx
