# Supplementary material for: Cross-reactive antibody against human coronavirus OC43 spike protein correlates with disease severity in COVID-19 patients: a retrospective study
Source: Emerg Microbes Infect. 2021 Apr 2;10(1):664–76. doi: 10.1080/22221751.2021.1905488 (PMC8023607; doi:10.1080/22221751.2021.1905488)
Supplement: Supplementary_materials.docx [file TEMI_A_1905488_SM4649.docx]

**Supplementary materials**

**Table S1****. Participant demographics information of recruited COVID-19 patients for four cohorts in this study**

|  | | Number of patients | | | | |
| --- | --- | --- | --- | --- | --- | --- |
|  | Cohort 1 | | Cohort 2 | Cohort 3 | Cohort 4 | Total |
| Mild/moderate | 72 | | 23 | 25 | 0 | 120 |
| Severe (Deaths) | 27 (10) | | 5(0) | 0 (0) | 192 (39) | 224 (49) |
| Total | 99 | | 28 | 25 | 192 | 344 |
|  |  | |  |  |  |  |
| One sample | 20 | | 17 | 25 | 14 | 76 |
| Two samples | 79 | | 11 | 0 | 42 | 132 |
| Three samples | 0 | | 0 | 0 | 58 | 58 |
| Four samples | 0 | | 0 | 0 | 66 | 66 |
| Five samples | 0 | | 0 | 0 | 12 | 12 |
| Total | 99 | | 28 | 25 | 192 | 344 |
|  |  | |  |  |  |  |
| Male/Female | 66/33 | | 19/9 | 9/16 | 115/77 | 209/135 |
| Age (Median) | 26–78 (49) | | 18–63 (41) | 25–73 (55) | 15–85 (58) | 15–85 (53) |

**Table S2.** **Number of plasma samples in four cohorts in this study**

| Disease severity | Cohort 1 | Cohort 2 | Cohort 3 | Cohort 4 | Total |
| --- | --- | --- | --- | --- | --- |
| Mild/moderate | 138 | 31 | 25 | 0 | 194 |
| Severe | 40 | 8 | 0 | 596 | 644 |
| Total | 178 | 39 | 25 | 596 | 838 |

**Table S3. Participant information of recruited COVID-19 patients for HCoV-OC43 RNA detection in this study**

|  | Cohort 1 | Cohort 2 | Cohort 3 | Cohort 4 | Total | |
| --- | --- | --- | --- | --- | --- | --- |
|  | Number of patients | | | | | |
| Mild/moderate | 12 | 23 | 25 | 0 | 60 | |
| Severe (Deaths) | 0 | 5(0) | 0 (0) | 192 (39) | 197 (39) | |
| Total | 12 | 28 | 25 | 192 | | 257 |
|  |  |  |  |  | |  |
|  | Number of samples | | | | | |
| Mild/moderate | 12 | 31 | 25 | 0 | | 68 |
| Severe | 0 | 8 | 0 | 596 | | 604 |
| Total | 12 | 39 | 25 | 596 | | 672 |

Figure S1. Temporal profiles of IgG against S proteins of HCoV-NL63, -229E, and -HKU1


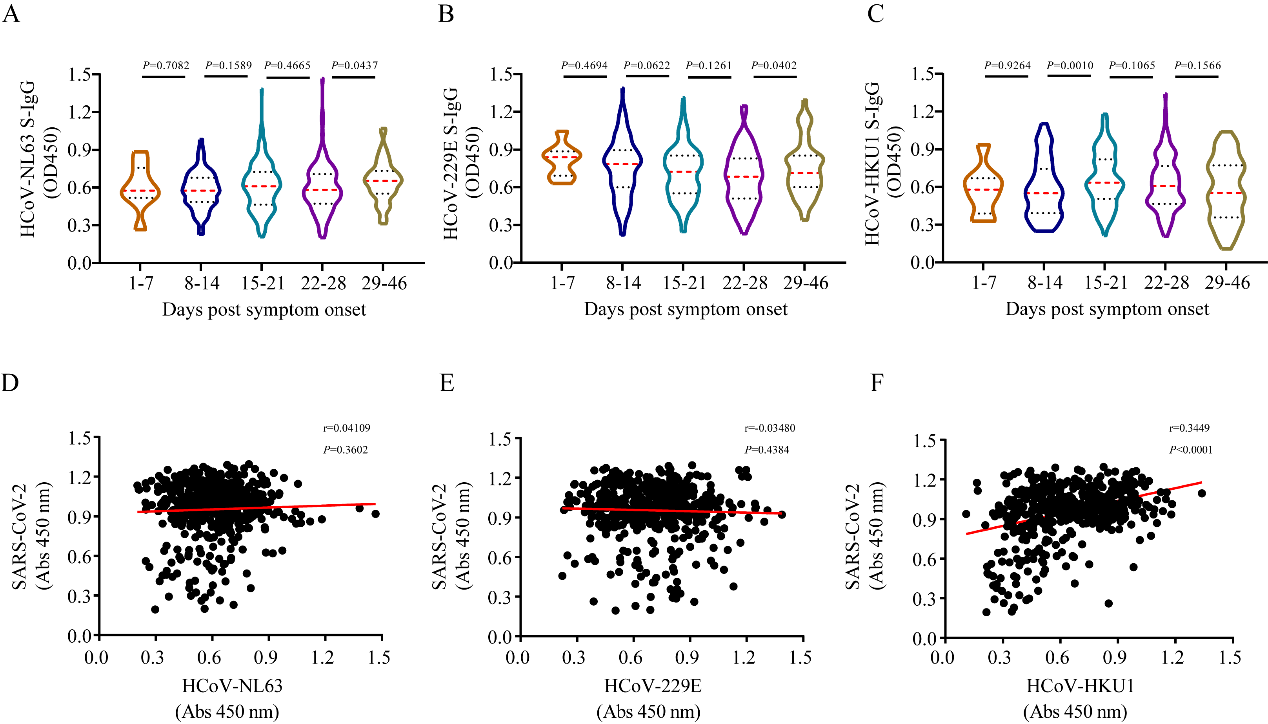


**(A–C)** Dynamic changes of HCoV-NL63 S-IgG (A), HCoV-229E S-IgG (B), and HCoV-HKU1 S-IgG (C) levels in COVID-19 patient’ plasma samples over time post symptom onset. Non-parametric Mann-Whitney test was used for comparison of antibody titers. Red and black dotted lines in violin denote the median and interquartile range of antibody titers, respectively. **(D–F)** Correlation between S-IgG antibody titers against HCoV-NL63 (A), -229E (B), -HKU1 (C) and SARS-CoV-2 S-IgG antibody titers in COVID-19 patients plasma samples. The correlations were assessed by Spearman’s rank correlation test.

**Figure S2. Temporal profiles of HCoV-OC43 S-IgG in consecutive samples from COVID-19 patients**


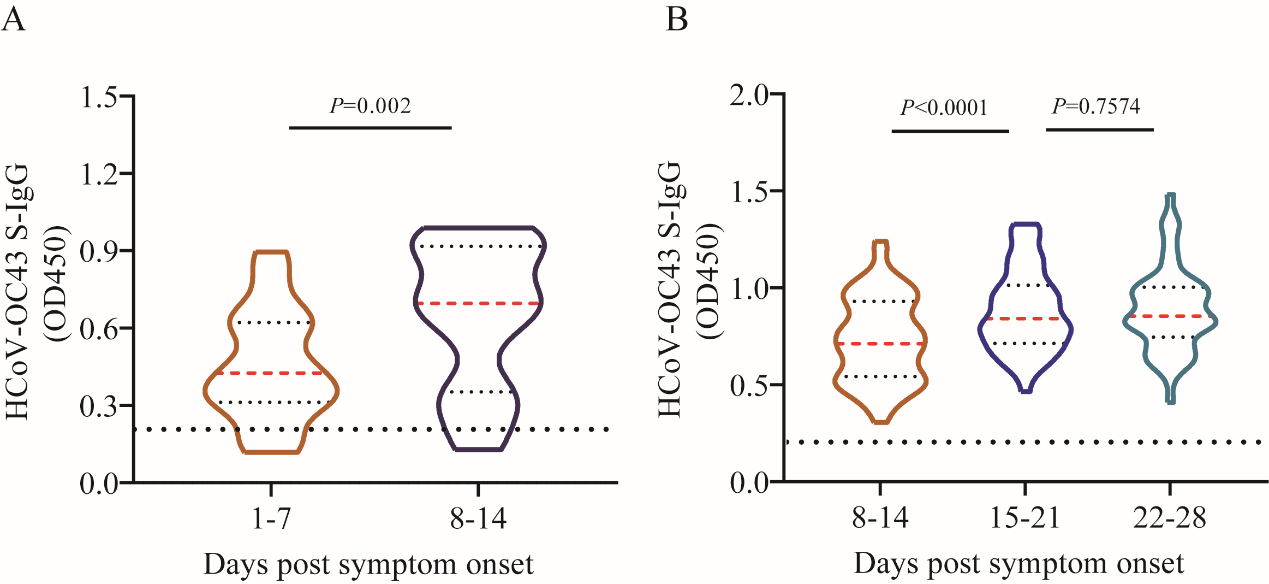


(A) 17 patients who had consecutive samples collected at days 1–7 and days 8 –14 post symptom onset (PSO). (B) 59 patients who had consecutive samples collected at days 8 –14, days 15 –21, and days 22–28 PSO. Two-tailed Wilcoxon matched-pairs signed-rank test was used for comparison of antibody titers.

**Figure S3. The amplification curve of HCoV-OC43 using throat swabs from COVID-19 patients.**


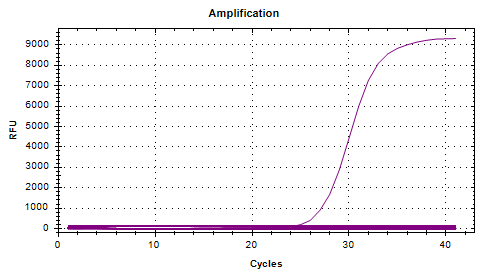


Samples were detected by a real-time RT-PCR (qRT-PCR) for HCoV-OC43. A respiratory tract sample positive for HCoV-43 was used as positive control.

**Figure S4. SARS-CoV-2 S-IgG antibody levels in unexposed children and adults, as well as COVID-19 patients in acute phase (days 1–10 post symptom onset).**


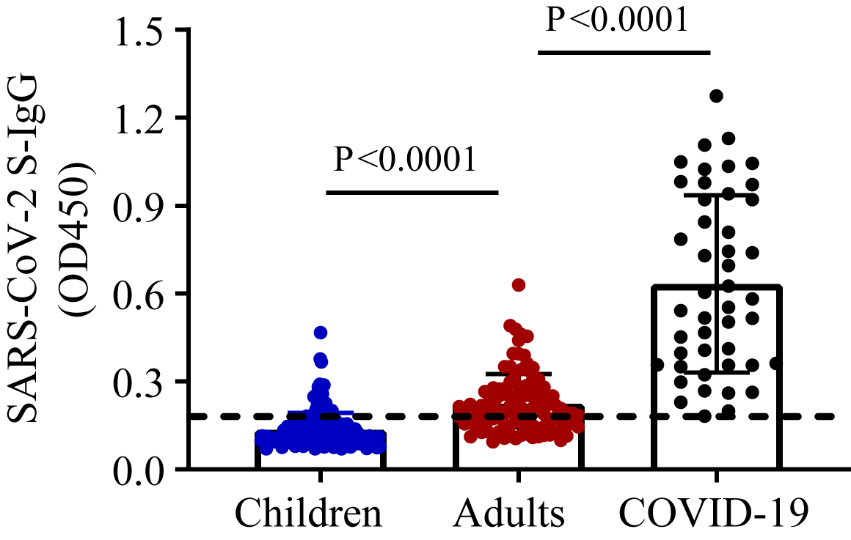


The plasma samples of unexposed children and adults were collected before 2019. The SARS-CoV-2 S-IgG antibody titers were evaluated using SARS-CoV-2 S-IgG ELISA assay. The dotted line represents the cut-off value of SARS-CoV-2 S-IgG ELISA. Non-parametric Mann-Whitney test was used for comparison of antibody titers.

**Figure S5. Antigenic relationship between HCoV-NL63, -229E, -HKU1 and SARS-CoV-2.**


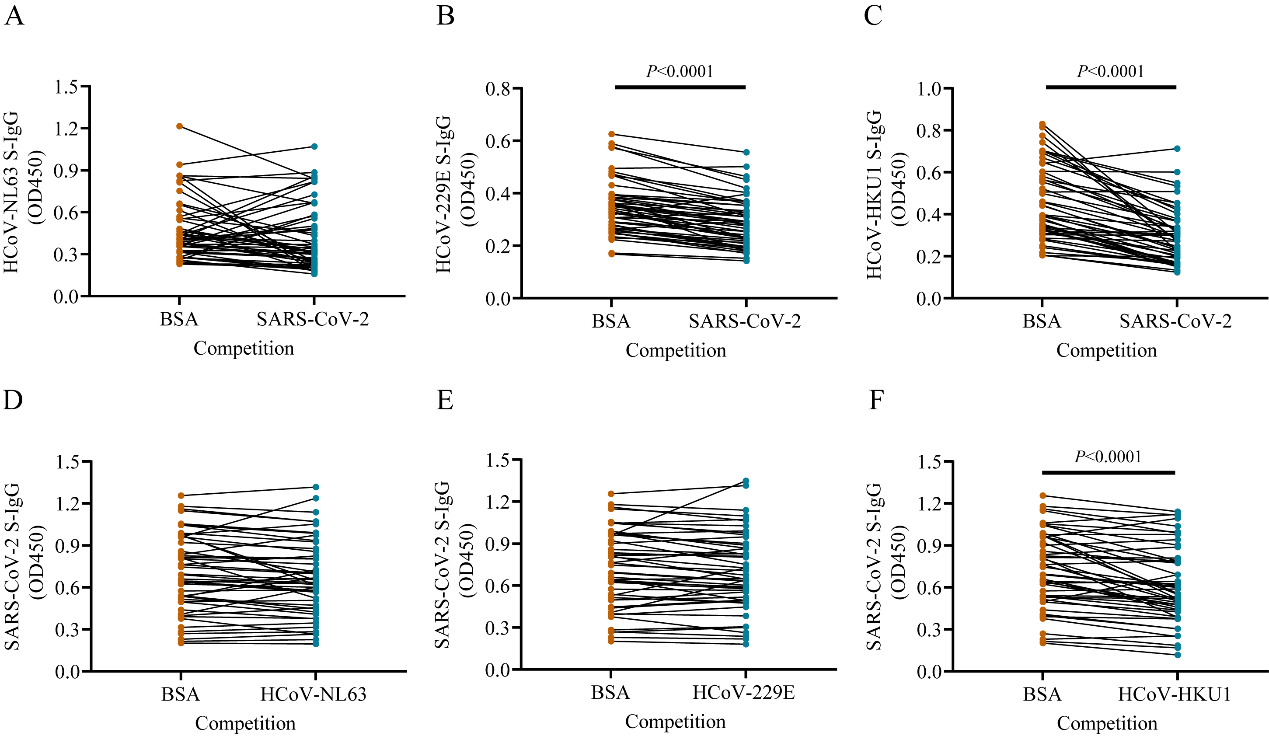


(A–C) Cross-reactivities between S-IgG antibodies against HCoV-NL63 (A), -229E (B), -HKU1(C) and SARS-CoV-2 S protein identified by competitive ELISA assays. (D–F). Cross-reactivities between S-proteins of HCoV-NL63(D), -229E(E), -HKU1 (F) and SARS-CoV-2 S-IgG identified by competitive ELISA assays. Two-tailed Wilcoxon matched-pairs signed-rank test was used for comparison of antibody titers.

**Figure S6. HCoV-OC43 S-IgG antibody levels in unexposed children and adults, as well as COVID-19 patients** **in acute phase (days 1–10 post disease onset).**


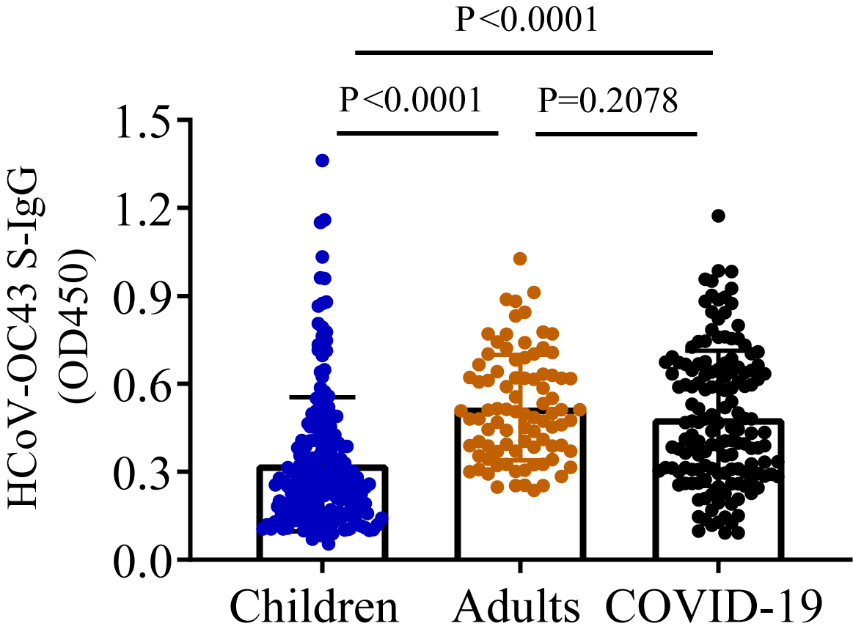


The plasma samples of unexposed children and adults were collected before 2019. The HCoV-OC43 S-IgG antibody titers were evaluated using HCoV-OC43 S-IgG ELISA assay. Student’s t-test was used for comparison of antibody titers. Non-parametric Mann-Whitney test was used for comparison of antibody titers.

**Figure S7.** **Cross-reactive HCoV-OC43 antibody titers correlate with disease severity in COVID-19 patients.**


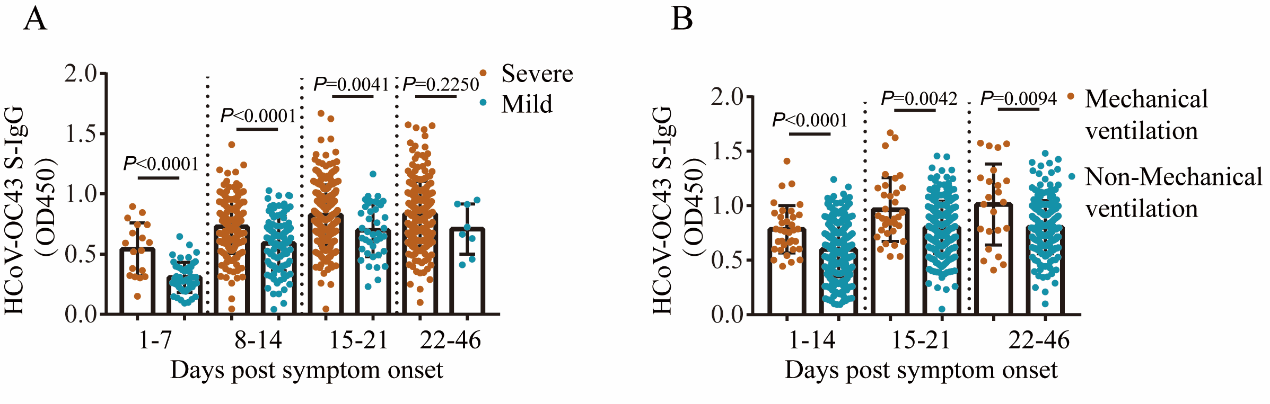


HCoV-OC43 S-IgG levels in COVID-19 patients with mild and severe symptom (A), and patients with mechanical ventilation and non-mechanical ventilation (B) over time post symptom onset. Non-parametric Mann-Whitney test was used for comparison of antibody titers.
